# Endurance training and pyruvate dehydrogenase kinase 4 (PDK4) inhibition combination is superior to each one alone in attenuating hyperketonemia/ketoacidosis in diabetic rats

**DOI:** 10.22038/ijbms.2024.79864.17305

**Published:** 2025

**Authors:** Hamed Rezaeinasab, Abdolhamid Habibi, Ramin Rezaei, Aref Basereh, Salva Reverentia Yurista, Kayvan Khoramipour

**Affiliations:** 1 Department of Exercise Physiology, Faculty of Physical Education and Sport Sciences, Shahid Chamran University, Ahvaz, Iran; 2 Department of Exercise Physiology, Central Tehran Branch, Islamic Azad University, Tehran, Iran; 3 Head of Research and Development Department, Islamic Republic of Iran Basketball Federation, Iran; 4 Department of Exercise Physiology, Faculty of Physical Education and Sport Sciences, Kharazmi University, Tehran, Iran; 5 Heart, Vascular and Thoracic Institute, Cleveland Clinic, Cleveland, OH, USA; 6 i+HeALTH Strategic Research Group, Department of Health Sciences, Miguel de Cervantes European University (UEMC), 47012 Valladolid, Spain

**Keywords:** Dietary supplements, Endurance training, Gene expression, Ketone bodies, Ketosis, Pyruvate dehydrogenase - kinase 4

## Abstract

**Objective(s):**

While ketone bodies are not the main heart fuel, exercise may increase their uptake. Objectives: This study aimed to investigate the effect of 6-week endurance training and Pyruvate dehydrogenase kinase 4 )PDK4( inhibition on ketone bodies metabolism in the heart of diabetic rats with emphasis on the role of Peroxisome proliferator-activated receptor-gamma coactivator PGC-1alpha (PGC-1α).

**Materials and Methods:**

Sixty male Wistar rats were divided into eight groups: healthy control group (CONT), endurance training group (TRA), diabetic group (DM), DM + EX group, Dichloroacetate (DCA) group, DM + DCA group, TRA + DCA group, and DM + TRA + DCA group. Diabetes was induced using streptozotocin (STZ). The animals in training groups ran on the treadmill for six weeks (30–50 min running at 20–30 m/min). After the training period, molecular markers for mitochondrial biogenesis and ketone metabolism were assessed in the heart. Circulating ß-hydroxybutyrate (ßOHB) and Acetylacetonate (AcAc) levels were also measured.

**Results:**

Our results showed that 6-week endurance training increased the cardiac expression of PGC-1α, 3-oxoacid CoA-transferase 1 (OXCT1), and Acetyl-CoA Acetyltransferase 1 (ACAT1) and reduced beta-hydroxybutyrate dehydrogenase1 (BDH1) expression (*P*≤0.05). In addition, exercise and DCA usage significantly decreased PDK4 gene expression, ßOHB, and AcAc blood levels (*P*≤0.05). Furthermore, the combination of 6-week endurance training and DCA supplementation led to more reduction in PDFK4 gene expression, ßOHB, and AcAc blood levels.

**Conclusion:**

Six-week endurance training and DCA supplementation could safely improve ketone body metabolism in the heart, ultimately reducing hyperketonemia/ketoacidosis in diabetic rats.

## Introduction

The heart uses a significant amount of energy. As it lacks energy reserves, it incessantly provides considerable amounts of adenosine triphosphate (ATP) to keep contractile function and ionic homeostasis (1-3). Bulks of ATP are generated in mitochondria via oxidative phosphorylation in which fatty acids and carbohydrates are the principal energy substrates in a manner that fatty acids contribute 50–75% of ATP production (1-3). However, this metabolic flexibility is damaged during insulin resistance, which may be seen in diabetic heart disease, where fatty acids are the only fuel source (4). It will increase ketone body (KB) production. KB accumulation may bring acidosis called ketoacidosis, which is a serious complication of diabetes (4). 

In healthy overnight fasted people, KB concentrations are about 50-200 μM. However, dramatic increases can be seen during continued fasting (~1 mM) or diabetic ketoacidosis (up to ~20 mM) (1). Beta-hydroxybutyrate dehydrogenase type1 (BDH1), succinyl-CoA:3-ketoacid coenzyme A transferase 1 (OXCT1), and Acetyl-CoA acetyltransferase 1 (ACAT1) are vital enzymes in KB metabolism which are located in mitochondria, with BDH1 being an inhibitor (1). Previous studies displayed a link between mutation of the genes encoding these enzymes and hyperketonemia/ketoacidosis in humans (2). Besides, OXCT1, as a knockout of the rate-limiting ketolytic enzyme, caused severe hyperketonemia/ketoacidosis in mice (3). However, mitochondrial biogenesis (i.e., increasing the size and number of mitochondria) could help to attenuate this condition by improving KB usage. 

Peroxisome proliferator-activated receptor-gamma coactivator (PGC)-1, including two alpha and beta isoforms acting on the biogenesis process, is the all-important regulator of mitochondrial biogenesis. The alpha isoform plays an important role (4). Studies have shown that PGC-1 expression could increase in response to physical activity/exercise, attenuating ketoacidosis in diabetes. However, the exact mechanism has not been understood yet. 

When fatty acids are metabolized, they use more oxygen per mole than glucose, which consequently enhances oxidative stress and reduces heart efficiency (5). The struggle between glucose and fatty acid oxidation is primarily controlled by the pyruvate dehydrogenase complex (PDC). Stimulating PDC could increase glucose usage and possibly reduce hyperketonemia/ketoacidosis. Dichloroacetate (DCA) and some of its derivatives are shown to inhibit pyruvate dehydrogenase kinase 4 (PDK4) (i.e., a PDC inhibitor), thus triggering PDC (6). Nives *et al*. (7) showed that PDK, in turn, is responsible for phosphorylating PDHE1α, thereby inhibiting PDC and diverting pyruvate metabolism towards lactate production (8). PDK comprises four distinct isoforms (PDK1–4), each possessing unique biochemical properties, distinct patterns of expression across different tissues, and specific functions. Notably, PDK4 exhibits elevated expression levels during periods of starvation and in cases of diabetes. While it is typically expressed at low levels, exceptions include the heart, skeletal muscle, and pancreatic islets, where it displays the highest basal activity among all PDK isoforms (9). Dysregulated PDK4, particularly its improper inhibition of PDC, has been associated with various pathological conditions, such as insulin resistance and type 2 diabetes (10, 11). Consequently, the pharmacological inhibition of PDKs has emerged as an appealing therapeutic approach for addressing these disorders (12). Building upon those findings, we hypothesized that 6-week endurance training and DAC supplementation could increase gene expression of PGC-1a, OXCT1, and ACAT1 but decrease BDH1 and PDK4 gene expression in the heart as well as serum levels of BOHB and AcAc. What could keep our study apart is the use of DAC and endurance training, two effective interventions together. We believe companioning these two could be more advantageous than each one alone. 

## Materials and Methods

The study was conducted following the guidelines provided by the Canadian Council on Animal Care (CCAC) or the Guide for the Care and Use of Laboratory Animals and agreed upon by the Ethical Committee at Ahmaz Jundishaour University of Medical Sciences (protocol #EE/97.24.3.70001/scu.ac.ir). A total of 60 male Wistar rats, aged eight weeks with an average weight of 200 ± 12g, were acquired from the Physiology Research Center at Ahvaz Jundishapur University of Medical Sciences in Iran. These rats were housed in Plexiglas cages under controlled conditions, maintaining a stable temperature of 23 ± 5 °C and a humidity level of 35 ± 5%. They were subjected to a 12-hr light/dark cycle. All rats had equal access to standard food and water throughout the study.

**Figure 1:**
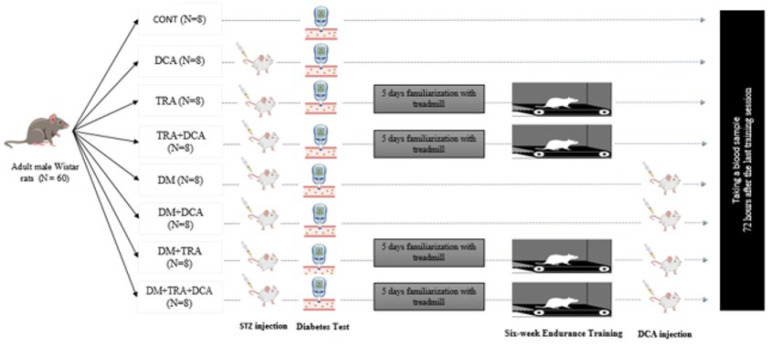
Experimental design. 60 male Wistar rats used in this study

After one week of acclimatization to the laboratory environment, the rats were carefully matched for their weights and then randomly divided into eight groups using a simple randomization approach. These groups included: healthy control group (CONT, n=8), healthy control + DCA (DCA, n=8), healthy + endurance training (TRA, n=8), healthy + endurance training + DCA (TRA+DCA, n=8), diabetic (DM, n=8), diabetic + DCA (DM+DCA, n=8), diabetic + endurance training (DM+TRA, n=8), and diabetic + endurance training + DCA (DM+TRA+DCA, n=8)(Figure 1). After a 12-hr fasting period, diabetes was induced by administering an intraperitoneal injection of streptozotocin solution (STZ) at a dosage of 50 mg per kilogram of body weight (50 mg/kg BW). This STZ solution was prepared by dissolving it in a 0.05 M citrate buffer with a pH of 4.5. 

Non-diabetic rats received the equivalent volume of citrate buffer (M = 0.05, pH = 4.5). Blood droplets accumulated on the tail were collected with a lancet 72 hr after the injection to measure glucose with a glucometer (Glucotrend 2, Roche Germany).

Rats were considered diabetic when their BGL was more than 300 mg/dl (13). To inhibit PDK4 activity in the myocardium, CONT+DCA, TRA+DCA, DM+DCA, and DM+TRA+DCA groups were given daily intraperitoneal injections of DCA 50 mg/kg body weight in a normal saline solution. The endurance training groups (DM-TRA, TRA, TRA+DCA, DM+TRA, and DM+TRA+DCA) were assigned to treadmill protocol for six weeks.

***DCA injection***

DCA was administered to the rats through intraperitoneal injections at 50 mg per kilogram of body weight, given at 24-hr intervals. The DCA was prepared by dissolving it in a methylcellulose 400 cP solution, which was combined with calcium gluconate for administration (14).

***Endurance training convention***

The rats were engaged in a six-week (five days/week) training schedule. Rats were attenuated to the treadmill (model LE7800, manufactured by Harvard Apparatus, France) at a 15 m/min speed for 20 min for seven days. The main training program commenced by adding the duration and speed progressively over six weeks so that in the final week, the speed reached 30 m/min, being equivalent to 75% of the animal’s maximum oxygen consumption, and the duration reached 50 min/day (15) (Table 1). Control groups were housed in sedentary conditions throughout the study.

Seventy-two hours after the last training session, rats were euthanized under anesthesia (by intraperitoneal injection of ketamine (90 mg/kg) and xylazine (90 mg/kg)). Eight milliliters of blood was taken from the right ventricle and anticoagulated with Ethylenediaminetetraacetic acid (EDTA). Blood samples were rapidly centrifuged, after which plasma was extracted. Plasma and myocardium were snap-frozen through submersion in liquid nitrogen and stored in the freezer at -80 °C for further analysis.

***Real-time PCR (RT-PCR)***

The researchers responsible for conducting the PCR tests and blood analyses were unaware of the study group assignments, ensuring a blind procedure. To extract mRNA, Isol-RNA was utilized. Approximately 100 milligrams of myocardium tissue were ground and homogenized in one milliliter of Isol-RNA Lysis Reagent (Qiagen, Germany). The homogenate was then centrifuged for 10 min at 12,000g (4 °C), resulting in the removal of the supernatant, which was subsequently transferred to a new microtube.

In the subsequent step, 200 μl of chloroform was added to the supernatant and vigorously mixed for 15 sec. Following this, the microtubes were again centrifuged for 15 min at 12,000g (4 °C). The aqueous phase was collected, and 600 μl of isopropyl alcohol was added. Another centrifugation at 12,000g (4 °C) was performed to extract total RNA.

The concentration of RNA and its purity were determined by assessing the OD260/280 ratio, with values between 1.8 and 2 considered acceptable purity. The synthesis of cDNA was carried out using TaKaRa’s cDNA synthesis kit (Fermentas, USA), following the manufacturer’s instructions. PCR reactions were conducted using AMPLIQON RealQ Plus 2x Master Mix Green High ROX (ABI, USA). The RT-PCR procedure involved an initial denaturation at 95 °C for 3 min, followed by 40 cycles, with denaturation at 95 °C for 45 min each cycle.

The GAPDH gene was employed as the reference gene to determine relative gene expression, and the melting curve analysis was used to verify product-specific amplification. The sequence details of the primers used in this study can be found in Table 2. Expression of the PDK4, PGC-1a, BDH1, OXCT1, and ACAT1 genes were measured by RT PCR method and were quantified using the method.

***Blood analysis***

βOHB and AcAc levels were quantified using beta a Hydroxybutyrate Assay Kit (Abcam 83390-United Kingdom) and Acetoacetate Assay Kit (abcam180875-United Kingdom) respectively, according to manufacturer’s instructions. 

***Statistical method***

The data are expressed as mean±standard deviations. Shapiro-Wilk and Levine’s tests were employed to evaluate the normal distribution of the data and the equality of variances. Differences between the groups were assessed using ANOVA followed by the Bonferroni test for *post hoc* comparisons. Significance was established at a threshold of *P*<0.05. All statistical analyses were conducted using IBM SPSS Statistics for Windows, version 21.

**Table 1:** Training protocol including one week of familiarization and six weeks of main training period. Male Wistar rats performed this protocol

7	6	5	4	3	2	1, familiarization	Week
30	28	28	24	24	20	15	Speed, m/min
50	50	40	40	30	30	20	Time, min
0	0	0	0	0	0	0	Incline

**Table 2:** Mouse (male Wistar rats)-specific primer pairs used for quantitative RT-PCR

bp	Reverse	Forward	
121	AAGTTCAACGGCACAGTCAAGG	CATACTCAGCACCAGCATCACC	Gapdh
78	TTGGACTTGAGGGTTAGGG	TCAGTGTTGAATGTGGTGAATGG	BDH1
196	TTCACCTTCCCCACACACTTC	TTCACCTTCCCCACACACTTC	OXCT1
243	TGCGAGTGGGAAATGAATGGG	GTGGTGGTGAAGGAAGATGAAGA	ACAT1
160	CTTCGCTGTCATCAAACAGG	AACAAGCACTTCGGTCATCC	PGC-1α
242	TGGATTGGTTGGCCTGGAAA	TATCGACCCCAACTGCGATG	PDK4

**Table 3:** Blood glucose of all groups (N=60 male Wistar rats) before and after 6-week

	CONT	DCA-CONT	TRA	DCA-TRA	DM	DM-TRA	DCA-DM	DCA-TRA-DM
BF blood glucose (mg/dl)	104±11	103±6	107±8	108±10	440±81*	412±77*	490±61*	472±58*
AF blood glucose (mg/dl)	110±14	106±6	103±4	111±07	431±69	274±32	308±52	178±28
BF-AF blood glucose difference	6	3	-4	3	9	-138^#^	-182^#^	-294^#@^

BF: before, AF: after. Healthy control (CONT), healthy control + DCA (CONT+DCA), healthy endurance training (TRA), healthy + endurance training + DCA (TRA+DCA), diabetes control (DM), diabetes control + DCA (DM+DCA), diabetes + endurance training (DM+TRA), and diabetes + endurance training + DCA (DM+TRA+DCA). * Shows significant difference with CONT. # Shows significance with DM. @ Shows significant difference with DM-TRA and DCA-DM

**Figure 2:**
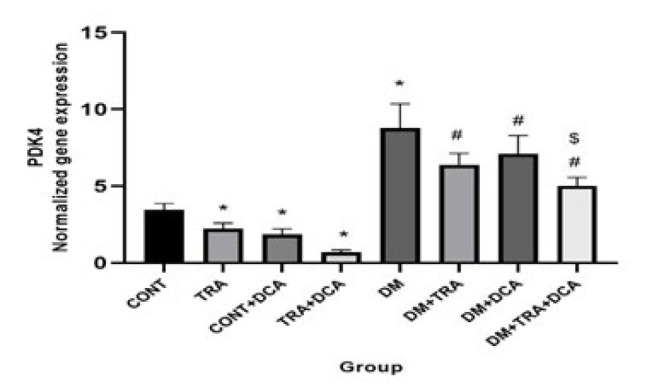
Normalized gene expression of PDK4 (mean±SD) in different groups (N=60 male Wistar rats)

Healthy control (CONT), healthy control + DCA (CONT+DCA), healthy endurance training (TRA), healthy + endurance training + DCA (TRA+DCA), diabetes control (DM), diabetes control + DCA (DM+DCA), diabetes + endurance training (DM+TRA), and diabetes + endurance training + DCA (DM+TRA+DCA)

Data are expressed as Mean ± SD. * significant differences compared to the CONT group (*P*≤0.01), # significant difference compared to the DM group (*P*≤0.01), and $ significant difference compared to DM-training and DM-DCA groups (*P*≤0.01).

**Figure 3:**
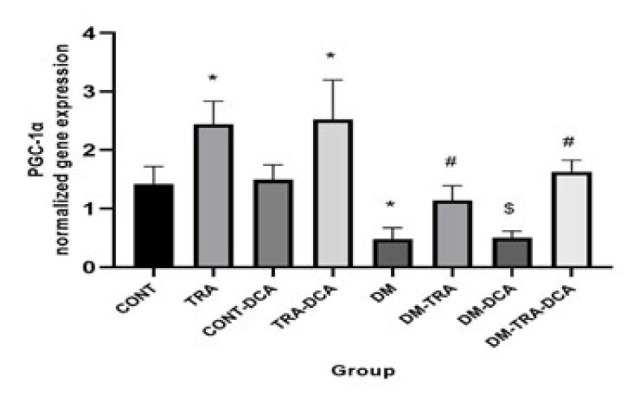
Normalized gene expression of PGC-1α (mean±SD) in different groups (N=60 male Wistar rats)

Healthy control (CONT), healthy control + DCA (CONT+DCA), healthy endurance training (TRA), healthy + endurance training + DCA (TRA+DCA), diabetes control (DM), diabetes control + DCA (DM+DCA), diabetes + endurance training (DM+TRA), and diabetes + endurance training + DCA (DM+TRA+DCA)

Data are expressed as Mean ± SD. * significant differences compared to the CONT group (*P*≤0.01), # significant difference compared to the DM group (*P*≤0.01), and $ significant difference compared to DM-training and DM-DCA groups (*P*≤0.01).

**Figure 4:**
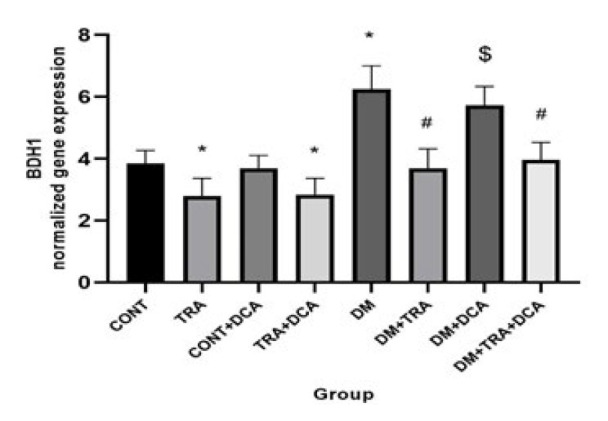
Normalized gene expression of BDH1 (mean±SD) in different groups (N=60 male Wistar rats)

Healthy control (CONT), healthy control + DCA (CONT+DCA), healthy endurance training (TRA), healthy + endurance training + DCA (TRA+DCA), diabetes control (DM), diabetes control + DCA (DM+DCA), diabetes + endurance training (DM+TRA), and diabetes + endurance training + DCA (DM+TRA+DCA)

Data are expressed as Mean ± SD. * significant differences compared to the CONT group (*P*≤0.01), # significant difference compared to the DM group (*P*≤0.01), and $ significant difference compared to DM-training and DM-DCA groups (*P*≤0.01).

**Figure 5:**
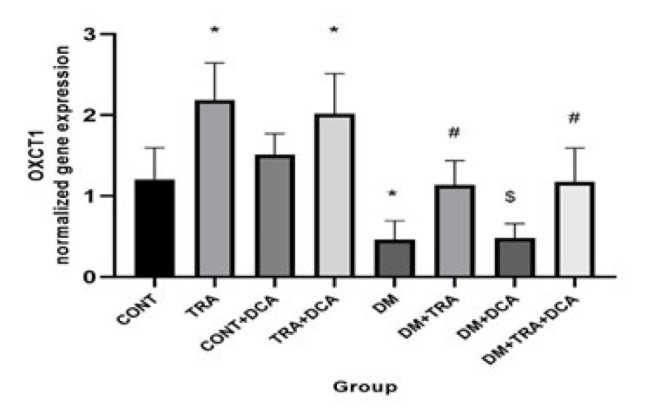
Normalized gene expression of OXCT1 (mean±SD) in different groups (N=60 male Wistar rats)

Healthy control (CONT), healthy control + DCA (CONT+DCA), healthy endurance training (TRA), healthy + endurance training + DCA (TRA+DCA), diabetes control (DM), diabetes control + DCA (DM+DCA), diabetes + endurance training (DM+TRA), and diabetes + endurance training + DCA (DM+TRA+DCA)

Data are expressed as Mean ± SD. * significant differences compared to the CONT group (*P*≤0.01), # significant difference compared to the DM group (*P*≤0.01), and $ significant difference compared to DM-training and DM-DCA groups (*P*≤0.01).

**Figure 6:**
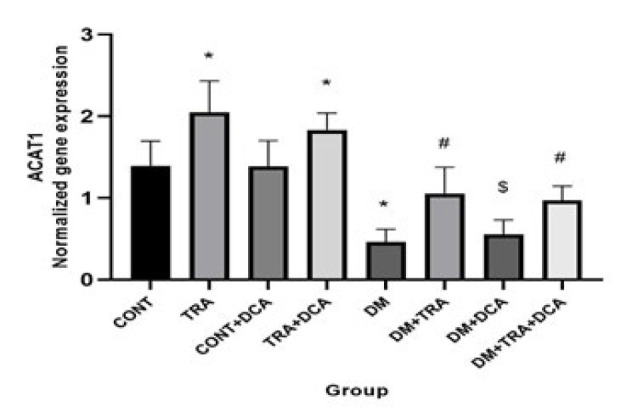
Normalized gene expression of ACAT1 (mean±SD) in different groups (N=60 male Wistar rats)

Healthy control (CONT), healthy control + DCA (CONT+DCA), healthy endurance training (TRA), healthy + endurance training + DCA (TRA+DCA), diabetes control (DM), diabetes control + DCA (DM+DCA), diabetes + endurance training (DM+TRA), and diabetes + endurance training + DCA (DM+TRA+DCA)

Data are expressed as Mean ± SD. * significant differences compared to the CONT group (*P*≤0.01), # significant difference compared to the DM group (*P*≤0.01), and $ significant difference compared to DM-training and DM-DCA groups (*P*≤0.01).

**Figure 7:**
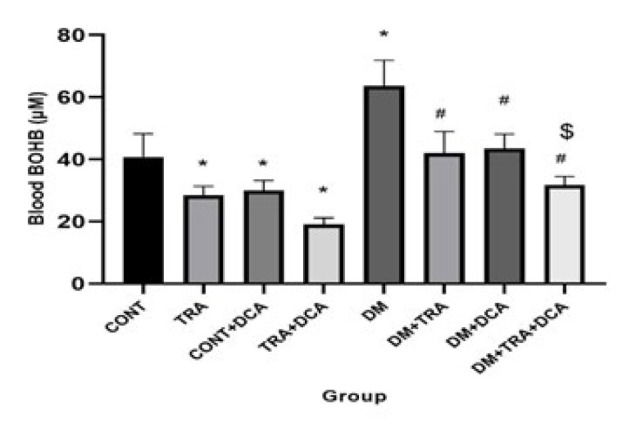
The Blood BOHB (µM) Levels (mean±SD) in different groups (N=60 male Wistar rats)

Healthy control (CONT), healthy control + DCA (CONT+DCA), healthy endurance training (TRA), healthy + endurance training + DCA (TRA+DCA), diabetes control (DM), diabetes control + DCA (DM+DCA), diabetes + endurance training (DM+TRA), and diabetes + endurance training + DCA (DM+TRA+DCA)

Data are expressed as Mean ± SD.* significant differences compared to the CONT group (*P*≤0.05), # significant difference compared to the DM group (*P*≤0.05), and $ significant difference compared to DM-training and DM-DCA groups (*P*≤0.05).

**Figure 8:**
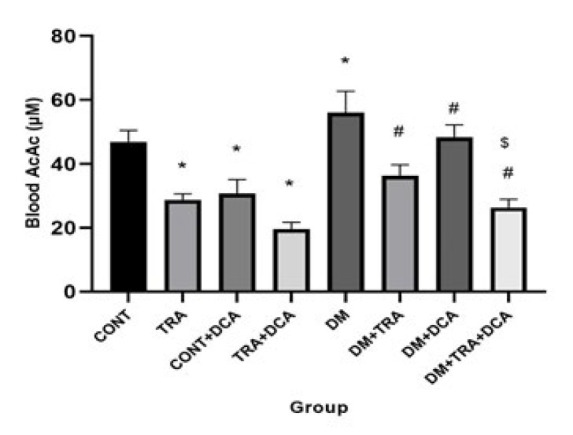
The Blood AcAc (µM) Levels (mean±SD) in different groups (N=60 male Wistar rats)

Healthy control (CONT), healthy control + DCA (CONT+DCA), healthy endurance training (TRA), healthy + endurance training + DCA (TRA+DCA), diabetes control (DM), diabetes control + DCA (DM+DCA), diabetes + endurance training (DM+TRA), and diabetes + endurance training + DCA (DM+TRA+DCA)

Data are expressed as Mean ± SD. * significant differences compared to the CONT group (*P*≤0.05), # significant difference compared to the DM group (*P*≤0.05), and $ significant difference compared to DM-training and DM-DCA groups (*P*≤0.05).

## Results

***Circulating blood glucose***

To check the diabetes induction methods, we measured blood glucose after diabetes induction (before (BF) blood glucose) in all groups (Table 3). Results showed that blood glucose was higher in DM-TRA (*P*=0.00), DM (*P*=0.001), DCA-DM (*P*=0.001), and DCA-TRA-DM (*P*=0.001) groups compared to the CONT group confirming diabetes induction. Blood glucose was measured again after 6-week endurance training and DCA injection (After (AF) blood glucose). We subtracted AF from BF blood glucose (BF-AF blood glucose difference) and compared the difference between groups. Blood glucose was decreased significantly in DM-TRA (*P*=0.001), DCA-DM (*P*=0.001), and DCA-TRA-DM (*P*=0.001) groups compared to DM group and in DCA-TRA-DM compered to DM-TRA (*P*=0.001) and DCA-DM (*P*=0.001) groups. All in all, these results suggest that both endurance training and PDH inhibition led to a decrease in blood glucose, and their combination is more effective in lowering blood glucose. 

***RT-PCR results***

PDK4 is an inhibitor of PDC; thus, its inhibition or down-regulation could redirect the metabolism to the Krebs cycle, increase glucose usage, and possibly reduce hyperketonemia/ketoacidosis. According to our results, PDK4 gene expression showed a significant difference between groups for exercise (F _(2,63)_ =88.32, *P*=0.001), DCA supplementation (F _(2,63)_ =100.23, *P*=0.001), and their interaction (F _(2,63)_ =89.63, *P*=0.001). Tukey *post hoc* test showed that PDK4 gene expression was lower (*P*≤0.05) and higher (*P*≤0.05) in the TRA, DCA, and DM groups compared to CONT, respectively confirming the positive effect of exercise and DCA and the negative effect of diabetes on PDK4 gene expression, in addition, PDK4 gene expression was lower in DM-TRA, DM-DCA, and DM-TRA-DCA groups compared to the DM group (*P*≤0.05). This data highlights the positive effect of exercise and DCA in diabetic rats. The lowest PDK4 gene expression was seen in the DM-TRA-DCA group (Figure 2), meaning that the combination of exercise and DCA is more effective than each sole. 

Increasing the mitochondria content could reduce hyperketonemia/ketoacidosis. The vital regulator of mitochondria biogenesis is PGC-1α. In addition, ACAT1 and OXCT1 are two enzymes that stimulate KB oxidation, and BDH1 is a suppressor of KB oxidation. Measuring all of these factors, we showed that there were significant differences in the PGC-1α (F_(2,63)=_2.81, *P*=0.041), ACAT1 (F_(2,63)=_13.36, *P*=0.001), and OXCT1 (F_(2,63)=_12.65, *P*=0.001) between groups for exercise. Tukey *post hoc* test showed that PGC-1α, OXCT1, and ACAT1 gene expressions were higher (P≤0.05) and lower (P=0.038) in the TRA and DM groups compared to CONT, respectively. This means that training and DCA injection effectively stimulated mitochondrial biogenesis and increased KB metabolism, but diabetes had reverse effects. In addition, their gene expressions were higher in the DM-TRA and DM-TRA-DCA groups compared to the DM group (P≤0.05), with the highest expression in the DM-TRA-DCA group, meaning that the combination of exercise and DCA is more helpful. But BDH1 gene expression showed significant difference for exercise (F_(2,63)=_68.99, *P*=0.000), DCA supplementation (F_(2,63)=_75.58, *P*=0.000), and their interaction (F_(2,63)=_81.23, *P*=0.000). BDH1 gene expression was lower in TRA and DM than in the CONT group. BHD1 gene expression was also lower in the DM-TRA and DM-TRA-DCA groups compared to the DM group. Considering the BHD1 data, we concluded that exercise and DCA suppress BHD1 gene expression with no advantage in their combination (Figure 3-6). 

***ELISA results***

ßHB and AcAc are the two main KBs in the blood. Our results showed a significant difference between ßHB and AcAc for exercise (F_(2,63)=_75.1, *P*=0.001), DCA injection, (F_(2,63)=_70.01, *P*=0.001) and their interaction (F_(2,63)=_89.3, *P*=0.001). A significant difference was between CONT with TRA, DCA, and DM groups (*P*≤0.05). In addition, the DM group showed significant differences with DM-TRA, DM-DCA, and DM-TRA-DCA groups with the lowest levels in DM-TRA-DCA (*P*≤0.05) (Figures 7 and 8). These data confirmed our RT-PCR data, showing that exercise and DCA effectively reduce KBs in blood circulation while their combination could be more effective. 

## Discussion

The present study was conducted to examine the role of PDK4 inhibition (using DCA) within 6-week endurance training on the expression of PGC-1α, PDK4, BDH1, OXCT1, and ACAT1 genes, as well as BOHB and AcAc plasma levels in the diabetic rats. Our results showed that while DM increased PDK4, BDH1, BOHB, and AcAc, DCA supplementation and exercise decreased them. Furthermore, PGC-1α, OXCT1, and ACAT1 decreased in the DM group but increased after training supplementation. In addition, a combination of DCA supplementation and exercise showed greater improvement than each alone in PDK4, BDH1, BOHB, and AcAc (Figures 3-6). 

 Fatty acids and glucose are recognized as the primary energy sources for a healthy heart, whereas lactic acids, ketone bodies, and amino acids play relatively minor roles (16, 17). In the case of a diabetic heart, glucose utilization is impaired due to insulin resistance, potentially leading to a predominant reliance on fatty acids for energy, which in turn results in the production of KBs (18). Despite elevated levels of circulating ketone bodies in diabetes, myocardial ketolytic activity is suppressed to prioritize and promote fatty acid oxidation during ketosis (19). Mizuno *et al*. (20) demonstrated that fasting individuals with diabetes exhibited higher total KB levels than non-diabetic individuals, a finding consistent with our results where ß-OHB and AcAc levels were elevated in the diabetic group compared to the control group. 

In line with other studies (5, 20, 21), our results showed that cardiac levels of PGC-1α are important for increased ketolytic capacity in response to endurance training, which could lead to decreased diabetic hyperketonemia. Cheng *et al*. (22) showed that PGC-1α plays a key role in modulating gene expression engaging in mitochondrial biogenesis and KBs oxidation, such as OXCT1, BDH1, and ACAT1. Endurance training reduces the levels of ATP and increases the intracellular calcium, which activates AMPK and CaMK pathways (23), leading to increased PGC-1α expression, which in turn increases OXCT1 and ACAT1 and decreases BDH1 gene expression (24). Intense aerobic exercise training is suggested to increase the expression of ketolytic enzymes OXCT1, BDH, and ACAT (25). Also, by improving insulin sensitivity in T2D, exercise works to prevent uncontrolled rates of ketogenesis (26), which was documented in our study by decreasing blood glucose in DM-TRA and DCA-TRA-DM groups.

Furthermore, DCA is a halogenated carboxylic acid known as a PDC activator (27) as it inhibits PDK4, resulting in aerobic metabolism enhancement, thus increasing glucose usage and lowering blood KBs (28). In line with our results, other studies (29, 30) reported that PDK4 inhibition by DCA injection reduced KBs in diabetic hearts and consequently decreased diabetic hyperketonemia.

## Conclusion

Taken together, endurance training and pyruvate dehydrogenase kinase 4 (PDK4) inhibition are superior to one alone in attenuating hyperketonemia/ketoacidosis in diabetic rats.

## Declaration

We have not used any AI tools or technologies to prepare this manuscript.
